# Watermelon Seed in Windpipe. Tracheotomy. Recovery

**Published:** 1894-09

**Authors:** W. L. Bullard

**Affiliations:** Columbus, Ga.


### *CORRESPONDENCE.*

---

### WATERMELON SEED IN WINDPIPE. TRACHEOTOMY. RECOVERY.

COLUMBUS, GA., July 30, 1894.

*Atlanta Medical and Surgical Journal:*

Some two weeks ago Mr. Brand, of Reynolds, Ga., by the advice ot his family physician, Dr. Rogers, brought to me his little girl, age three and half years old, with a foreign body in the trachea. Accident had happened some five or six days previously. Microscopically patient showed evidence of suffering from the usual mental suspense found in cases with foreign body in air passage. The child had a croupy cough and almost an incessant one. The breathing was somewhat labored, with symptoms of no little venous congestion. The *corpus alien* could be heard on auscultation to be free and movable with each respiration, which accounted for the incessant cough, and also removed any apprehension of its impaction. The little one was very much excited and uncontrollable, which rendered a laryngoscopic examination an impossibility. Being satisfied that the body had passed beyond or below the larynx, I also knew that an intra-laryngeal extraction would be an impossibility. So tracheotomy was advised, which was consented to, and with the assistance of Drs. Rogers, of Reynolds, Ga., Grimes, McDuffie and Griggs, of this city, the child was chloroformed, and the low operation performed in the usual way; only we were very careful not to wound any of the distended veins (which are always found when the air passages are obstructed). To do this prolonged the operation, yet we were gratified to know that the loss of blood did not exceed a tablespoonful, possibly. When the trachea was reached it was hooked with a tenaculum, raised and three rings easily incised, when with tracheal hooks the incision was pulled apart, and to our gratification, a watermelon seed was with great force ejected with the first respiratory movement. The after treatment was conducted as is usual in such cases, save that only one suture (worm-gut) was put in the trachea, and a shred of iodoform gauze left in lower portion of skin wound as drainage. The whole dressed with iodoform gauze. After an hour or two (the patient was operated on at my office) patient was carried to the hotel and the wound healed in five days, without any accident, at which time the little one was taken home by its mother, with a heart full of gratitude, etc.

W. L. BULLARD, M.D.

---

### CHOLERA INFANTUM.

The hygienic management of infants is of the utmost importance, and every true physician will give each mother who seeks his advice in regard to the care of those entrusted to her, full instruction in regard to the following points:

1. Pure air to breath.
2. Proper diet at regular intervals.
3. Plenty of water which has been boiled to drink.
4. A clean bottle, if bottle fed, every time it takes its food.
5. Absolute cleanliness in person and surroundings.
6. A chance for the nervous system to be developed without being over-excited.

Physicians coincide in their views regarding the treatment of the summer diarrhea of infants and children to a degree that enables it to be thus briefly summarized: Diet, emptying the alimentary tract, antisepsis. For the antiseptic treatment, listerine alone, or listerine, aqua cinnamon and glycerine, or listerine, bismuth and mistura creta, will meet many requirements of the practitioner during the summer months.

According to a reliable authority the following treatment has been adopted in most cases and with happy results: Stop milk and other food and give only Mellin's food or one of those akin to it. Give one of the following powders every hour till the stools become less offensive in odor and more natural in consistency:

℞ Salol.......................................................gr. j.
Zinci sulpho-carbolat.................................gr. ss.
M.

